# Age, sex, and apolipoprotein E isoform alter contextual fear learning, neuronal activation, and baseline DNA damage in the hippocampus

**DOI:** 10.1038/s41380-023-01966-8

**Published:** 2023-02-02

**Authors:** Sydney Weber Boutros, Benjamin Zimmerman, Sydney C. Nagy, Vivek K. Unni, Jacob Raber

**Affiliations:** 1 Department of Behavioral Neuroscience, OHSU, 3181 SW Sam Jackson Park Rd, Portland, OR 97239 USA; 2 Advanced Imaging Research Center, OHSU, 3181 SW Sam Jackson Park Rd, Portland, OR 97239 USA; 3 Helfgott Research Institute, NUNM, 2201 SW First Avenue, Portland, OR 97201 USA; 4 Beckman Institute for Advanced Science and Technology, University of Illinois at Urbana-Champaign, 405 N, Matthews Avenue, Urbana, IL 61801, USA; 5 Department of Neurology, OHSU, 3181 SW Sam Jackson Park Rd, Portland, OR 97239 USA; 6 Jungers Center for Neurosciences Research, OHSU; and OHSU Parkinson Center, 3181 SW Sam Jackson Park Rd, Portland, OR 97239 USA; 7 Departments of Psychiatry and Radiation Medicine, OHSU, 3181 SW Sam Jackson Park Rd, Portland, OR 97239 USA; 8 Division of Neuroscience, ONPRC, 505 NW 185th Ave, Beaverton, OR 97006 USA; 9 Present Address: Department of Psychological Sciences, Boise State University, 2133 W Cesar Chavez Ln, Boise, ID 83725 USA

**Keywords:** Neuroscience, Psychology

## Abstract

Age, female sex, and apolipoprotein E4 (E4) are risk factors to develop Alzheimer’s disease (AD). There are three major human apoE isoforms: E2, E3, and E4. Compared to E3, E4 increases while E2 decreases AD risk. However, E2 is associated with increased risk and severity of post-traumatic stress disorder (PTSD). In cognitively healthy adults, E4 carriers have greater brain activation during learning and memory tasks in the absence of behavioral differences. Human apoE targeted replacement (TR) mice display differences in fear extinction that parallel human data: E2 mice show impaired extinction, mirroring heightened PTSD symptoms in E2 combat veterans. Recently, an adaptive role of DNA double strand breaks (DSBs) in immediate early gene expression (IEG) has been described. Age and disease synergistically increase DNA damage and decrease DNA repair. As the mechanisms underlying the relative risks of apoE, sex, and their interactions in aging are unclear, we used young (3 months) and middle-aged (12 months) male and female TR mice to investigate the influence of these factors on DSBs and IEGs at baseline and following contextual fear conditioning. We assessed brain-wide changes in neural activation following fear conditioning using whole-brain cFos imaging in young female TR mice. E4 mice froze more during fear conditioning and had lower cFos immunoreactivity across regions important for somatosensation and contextual encoding compared to E2 mice. E4 mice also showed altered co-activation compared to E3 mice, corresponding to human MRI and cognitive data, and indicating that there are differences in brain activity and connectivity at young ages independent of fear learning. There were increased DSB markers in middle-aged animals and alterations to cFos levels dependent on sex and isoform, as well. The increase in hippocampal DSB markers in middle-aged animals and female E4 mice may play a role in the risk for developing AD.

## Introduction

Sporadic Alzheimer’s disease (AD) comprises more than 90% of AD diagnoses [1]. Age and female sex are the two highest risk factors for development of late onset AD (LOAD) [2, 3]. Additionally, risk to develop post-traumatic stress disorder (PTSD) is higher in women than men [4]. Apolipoprotein E (apoE) isoform is the next greatest predictor for LOAD [5, 6]. ApoE exists in three major human isoforms: E2, E3, and E4. Compared to E3 carriers, E4 carriers have a higher risk and E2 carriers a lower risk to develop LOAD [6]. This relative protection/risk for LOAD is ethnicity-dependent: the E4-LOAD risk is seen in White and Japanese populations but absent in Native American and non-white Hispanic populations and variable in Black populations [6–8]. While E2 is relatively protective against LOAD, it is associated with higher rates and greater severity of PTSD [9–11]. Evidence in both people and mice shows that E2 carriers have poorer outcomes in melanoma than E4 carriers (increased tumor size, metastasis, and reduced survival) [12]. How the apoE isoforms lead to differential and specific disease risk or protection is unclear.

Importantly, sex and apoE affect cognition throughout life. For example, 7–10-year-old female E4 carriers perform worse on visual and spatial recognition tasks than female E3 carriers [13]. However, this effect is not seen in males. In young adults, E4 carriers show increased brain glucose metabolism [14]. After AD diagnosis, women show a faster cognitive decline than men despite similar amyloid β levels [15, 16]. This sex difference in cognitive decline is possibly due to increased oxidative stress and pro-inflammatory markers in aging women [17–19]. Investigations looking at brain activation at rest and during learning and memory tasks have shown apoE-dependent differences in healthy middle-aged people in the absence of cognitive differences, with E4-carriers displaying greater blood oxygen levels during learning trials compared to age- and sex-matched E3 carriers [20]. These studies together suggest that E4 carriers have increased energy usage at young ages.

In rodents, immediate early genes (IEGs)—such as cFos—are used as markers of neuronal activation, as they have low baseline levels, are rapidly expressed upon stimulation, and return to baseline levels within a few hours [21, 22]. IEGs are important for synaptic plasticity, learning, and memory [21–26]. Interfering with typical IEG expression impairs memory retrieval [27] and induced IEG expression can create false memories [28] in rodents. ApoE-dependent differences in expression of IEGs are relatively unexplored. One study reported that E4 mice on a high fat diet show less IEG expression in the hippocampus than E3 mice [29]; another found apoE-dependent changes in phosphorylation of cFos (an IEG) in induced pluripotent stem cells (iPSCs) [30].

The mechanisms that lead to IEG expression are unclear. Recently, evidence has emerged for a role for DNA double strand breaks (DSBs) in IEG expression. Stimulation of hippocampal neurons in vitro leads to DSBs on the transcription start sites of IEGs [31]. Exposure of 4–6 month old C57Bl/6J wild-type (WT) mice to a novel environment leads to a transient increase in γH2Ax—a DSB repair marker—in relevant brain regions [32], and WT mice show increased hippocampal DSBs after fear learning [31]. Tight regulation of DSBs and their proper repair is likely important for this mechanism. Young mice carrying familial dominant mutations for AD have higher baseline levels of γH2Ax at baseline and fail to return to baseline levels 24 hours after stimulation [32]. Increased γH2Ax and 53BP1 (another DSB repair marker) are also observed in post-mortem tissue of patients with mild cognitive impairment (MCI) and AD [33], suggesting that dysregulation of DSBs might be an early, driving factor in age-related cognitive decline and dementia.

Contextual fear conditioning is a well-studied learning test with clearly defined neuroanatomy [34–36]. cFos expression increases in specific brain regions of rodents after fear conditioning and is dependent on the paradigm used [37, 38]. The hippocampus is particularly important for contextual fear conditioning. Increases are seen in the hippocampus following contextual fear conditioning [38], and inhibition of cFos in the CA1 impairs contextual recall [39]. Beyond the hippocampus, many regions have been defined that contribute to different aspects of fear encoding, including somatosensation (such as the locus coeruleus), contextual encoding (such as the entorhinal cortex), fear integration (the amygdalar nuclei), and fear expression (such as the periaqueductal gray) [34]. These rodent regions mirror those observed in human functional magnetic resonance imaging (fMRI) during fear conditioning [40].

There are apoE isoform differences in anxiety-like and fear learning and memory in human apoE targeted replacement (TR) mice, which express the human isoforms under control of the murine apoE promoter. E2 mice display impaired fear extinction [41], and E4 mice show increased anxiety-like behavior [42]. Moreover, E2 mice show alterations in the endocrine system and behavioral measures following exposure to trauma, which are potentially related to the higher PTSD clinical scores and blunted cortisol levels in E2-carrying combat veterans and refugees [9, 11].

Altogether, early-life differences in IEG signaling and DSB regulation might contribute to the apoE-specific risks for LOAD and PTSD. Here, we used young (3 months) and middle-aged (12 months) male and female apoE TR mice to assess DSBs and IEG expression at baseline and following contextual fear conditioning. Due to increased risk for females to develop AD and PTSD, we also investigated possible early changes using whole-brain cFos imaging in young female apoE TR mice [3] following fear learning. We assessed brain-wide alterations in neuronal activation [43] and co-activation across regions similar to functional MRI analyses [44, 45]. We hypothesized that each apoE isoform would show distinct patterns of cFos activation and DSB formation and repair, and that alterations would become more pronounced with aging.

## Materials & methods

### Mice

We bred young (3 months old) and middle-aged (12 months old) male and female human apoE TR mice, originally generated by Sullivan et al. [46–48]. A total of *n* = 306 mice were used: experiment 1 (whole brain imaging) *n* = 60; experiment 2 (cFo + 53BP1 immunohistochemistry) *n* = 126; and experiment 3 (ChIP-ddPCR) *n* = 123. Mice were randomly assigned to groups.

**Fig. 1:**
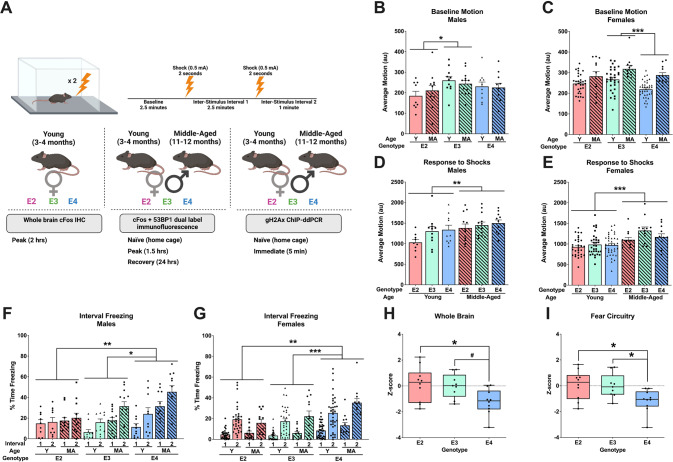
Effects of age and apoE isoform on fear learning. **A** Schematic of the experimental design. **B** Average baseline motion in males during the initial 2 min of fear conditioning. There was a main effect of apoE isoform (*p* = 0.037, *F*(2,60) = 3.480), driven by E2 vs. E3 (*p* = 0.042). There was no effect of age (*p* = 0.927) and no interaction (*p* = 0.571). **C** Average baseline motion in females. There was a main effect of apoE isoform (*p* = 0.006, *F*(2,130) = 5.301), driven by E3 vs. E4 (*p* < 0.001). Middle aged mice moved more than young mice (*p* < 0.001); there was no interaction (*p* = 0.403). **D** Average motion during the two shocks in males. Middle aged mice moved more than young mice (*p* = 0.0055, *F*(1,60) = 8.246). There were no apoE isoform differences (*p* = 0.068) and no interaction (*p* = 0.458). **E** Average motion during the two shocks in females. Middle aged mice moved more than young mice (*p* < 0.0001, *F*(1,130) = 23.25). There were no apoE isoform differences (*p* = 0.070) and no interaction (*p* = 0.317). **F** Percent time freezing during the ISIs in males. There was an effect of time (*p* < 0.001, *F*(1,60) = 22.842), isoform (*p* = 0.005, *F*(2,60) = 5.90), age (*p* < 0.001, *F*(1,60) = 19.087), and a time-by-isoform interaction (*p* = 0.043, *F*(2,60) = 3.318). E4 mice froze more than E2 (*p* = 0.008) and E3 (*p* = 0.014) mice. **G** Percent time freezing during the ISIs in females. There was an effect of time (*p* < 0.001, *F*(1,130) = 165.030), isoform (*p* < 0.001, *F*(2,130) = 10.258), and a time-by-isoform interaction (*p* = 0.049, *F*(2,130) = 3.087). E4 mice froze more than E2 (*p* = 0.002) and E3 (*p* < 0.001) mice. **H** Z-scores of cFos-positive cells in the entire brain of young, female mice. There was a main effect of apoE isoform (*p* = 0.020, *F*(2,26) = 4.573), driven by E2 vs. E4 (*p* = 0.0319) and a trend in E3 vs. E4 (*p* = 0.0664). **I** Z-scores of cFos-positive cells in all regions involved in contextual fear learning in young, female mice. There was a main effect of apoE isoform (*p* = 0.0172, *F*(2,26) = 4.773), driven by E2 vs. E4 (*p* = 0.0270) and E3 vs. E4 (*p* = 0.0434) mice. Behavioral data are presented as mean ± SEM. cFos immunoreactivity data are presented as minimum to maximum boxplots. Each point represents an individual animal. ^**#**^*p* < 0.07, *p < 0.05, **p < 0.01, ****p < 0.0001.

Experiment 1 involved 1 behavioral condition (fear training); experiment 2 involved 3 behavioral conditions (behaviorally naïve, fear training, or fear training + recovery); experiment 3 involved 2 behavioral conditions (behaviorally naïve or fear training; Fig. 1A). All mice were singly housed 4 days prior to testing and provided extra nesting material. Food and water were provided *ad libitum* and lights were on a standard 12 h light: dark cycle.

All animal procedures were reviewed and approved by the OHSU IACUC and in accordance with AAALAC standards. All procedures followed the ARRIVE guidelines. Researchers were blinded to groups throughout the duration of experiments until all data were analyzed.

### Contextual fear conditioning

We used a standard 2-shock contextual fear conditioning paradigm, as described previously [41]. Mice received two 0.5 mA shocks over the course of 6 min in plexiglass chambers with a shock grid (Med Associates, St. Albans, Vermont).

### Tissue collection & whole-brain imaging

Experiment 1: Two hours after fear conditioning, mice were intracardially perfused to capture peak cFos levels [49, 50]. Brains were stored overnight in 4% PFA, then switched to PBS and sent to Certerra, Inc. (acquired by Certego Therapeutics) for whole brain imaging as previously described [43].

Experiment 2: Mice were intracardially perfused 1.5 h after fear conditioning (the overlap between peak cFos expression and 53BP1 [50, 51]), 24 h after fear conditioning, or without ever experiencing fear conditioning (behaviorally naïve). Brains were switched to cryopreserve after 24 h.

Experiment 3: Mice were euthanized by cervical dislocation and decapitation either 5 min after fear conditioning [52] or behaviorally naïve. The hippocampus and cortex were dissected and flash frozen in liquid nitrogen, then stored at −80 °C until use.

### Dual-label immunofluorescence & microscopy

Immunofluorescence was done on free-floating 40 µm sections [53, 54] using primary antibodies against 53BP1 (Bethyl Labs, #A300-272A) and cFos (PhosphoSolutions, #309-cFOS), secondary antibodies (AlexaFluor647, Invitrogen, #A-21245; and AlexaFluor488, Invitrogen, #A-11001) and a DAPI (Sigma D9542) counterstain. Sections were slide mounted with CitiFluor CFMR2 Antifade Solution and sealed with Biotium CoverGrip Coverslip Sealant.

Z-stack images of the CA3 and CA1 (3 sections/animal) were taken using a Zeiss LSM 980 with Airyscan 2 at 63x zoom. The number of cells with 53BP1 foci, cFos, and co-localized signal were manually counted using ImageJ (NIH, Bethesda, MD).

### Chromatin Immunoprecipitation & digital droplet PCR

Chromatin immunoprecipitation (ChIP) was done with a ThermoFisher MAGnify Kit (ThermoFisher, #492024) as previously described [55] on hippocampal tissue using a γH2Ax antibody (Cell Signal, #9718). Concentrations for ChIP and input samples were measured using the Qubit dsDNA High Sensitivity kit (ThermoFisher).

Digital droplet PCR (ddPCR) was used to assess cFos, Npas4, and BDNF expression [56]. Samples were prepared according to the BioRad QX200™ Droplet Digital™ PCR System manual (BioRad, Hercules, CA).

### Statistical analysis

All data were first analyzed for normality. We implemented Bonferroni corrections for all *post hoc* tests. Due to the repeated effects of and interactions with sex, we split males and females for all analyses.

For fear conditioning, average motion and percent time freezing were analyzed using an ANOVA. For learning, percent time spent freezing during the ISIs was analyzed with a repeated-measures ANOVA.

The number of cFos+ cells, 53BP1 + cells, and cFos-53BP1 + cells in the CA1 and CA3 were analyzed with a multi-way ANOVA. The concentration of cFos, Npas4, and BDNF (measured in copies/µL) was normalized to total DNA and analyzed with an ANOVA. To determine relative enrichment, cFos and NPAS4 concentrations were compared to BDNF levels.

For whole-brain analysis, ClearMap was applied to collected light-sheet images to segment brain regions and count number of cFos+ cells [57]. We first analyzed the raw number of cFos+ cells throughout the brain. We discovered significant differences between cohorts, likely due to the sensitive nature of IEGs. As such, we transformed the cFos counts into Z-scores, normalized to the E3 animals in each cohort.

We then proceeded to analyze differences in cFos activation across the brain. We identified brain regions known to be important for contextual fear learning [34, 35] and organized them into 4 distinct subcategories: somatosensation, contextual encoding, fear integration, and fear expression (supplemental table 1). We then used repeated-measures ANOVAs to identify regional and isoform-dependent differences. To localize regional differences, we subsequently used MANOVAs with Bonferroni corrections.

We have previously used this type of data as an indirect measure of connectivity [44, 45]. Here, we calculated the Pearson’s coefficient for all fear-related brain regions, built correlation matrices, and compared the matrices, as previously described [58]. This method is sensitive to small numbers of pairwise differences, making the test appropriate for examining differences in gene expression [59] or neuronal connectivity [45]. This produces a test statistic, $\hat T$; the *p*-value is determined by counting the number of multiplier bootstraps larger than the test statistic [60]. This was shown to be appropriate even in cases where the number of dimensions is greater than the number of subjects [58].

## Results

### ApoE isoform-dependent differences in fear learning and whole-brain cFos expression

We first assessed if apoE isoform affects contextual fear learning (Fig. 1A). Prior to any shocks, we detected a difference in baseline motion between the distinct apoE isoforms in both males (*p* = 0.037, Fig. 1B) and females (*p* = 0.006, Fig. 1C). E3 males moved more than E2 males (*p* = 0.042) and E3 females moved more than E4 females (*p* < 0.001). Additionally, middle-aged females moved more than young females (*p* < 0.001). There were no apoE-dependent differences in average motion during the shocks, though middle-aged mice moved more than young mice in both males (*p* = 0.006, Fig. 1D) and females (*p* < 0.001, Fig. 1E).

The percent time freezing during the inter-stimulus intervals (ISIs), a measure of fear learning, was dependent on apoE isoform (males: *p* = 0.005, Fig. 1F; females: *p* < 0.001, Fig. 1G). Male and female E4 mice froze more during the ISIs than either E2 (*p* = 0.008, *p* = 0.002, respectively) or E3 mice (*p* = 0.014, *p* < 0.001, respectively). Additionally, middle-aged male mice froze more than young male mice (*p* < 0.001); middle-aged female mice trended towards freezing more than young female mice (*p* = 0.071). While all mice displayed an increase in freezing from ISI 1 to ISI 2 (*p* < 0.001, both sexes), this pattern was distinct in the isoforms as shown by a time-by-isoform interaction (males: *p* = 0.043; females: *p* = 0.049).

Following fear conditioning, we assessed brain activation via cFos immunoreactivity across the whole brain and in regions known to contribute to contextual fear learning in young female mice (Supplemental Table 1). There were apoE isoform differences in the global magnitude of cFos activation (*p* = 0.020, Fig. 1H), with E4 mice having less cFos immunoreactivity than E2 mice (*p* = 0.0319) and trended towards having less cFos immunoreactivity than E3 mice (*p* = 0.0664). We next focused our analysis on regions important for contextual fear learning. Again, there was an overall difference dependent on apoE isoform (*p* = 0.0172; Fig. 1I). *Post hoc* testing revealed that E4 mice had less cFos immunoreactivity in all fear learning regions compared to E2 (*p* = 0.0270) and E3 mice (*p* = 0.0434).

### ApoE isoform-dependent differences in magnitude of cFos activation in fear circuitry of young female mice

To clarify differences between the apoE isoforms in brain activation, we organized brain regions involved in contextual fear learning into distinct categories: somatosensation, contextual encoding, fear integration, and fear expression (Supplementary Table 1).

**Fig. 2:**
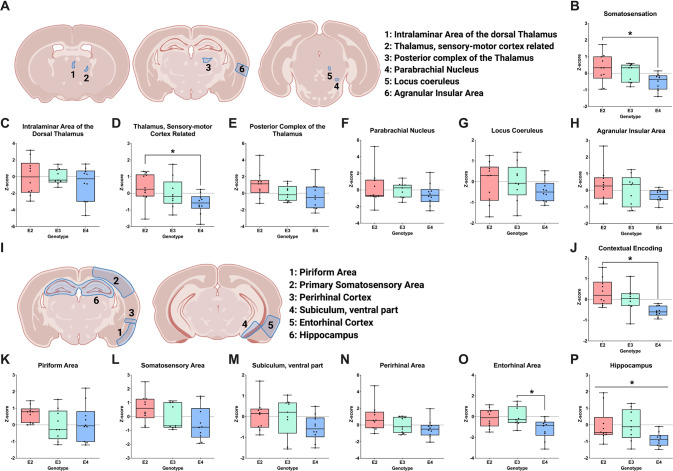
cFos immunoreactivity in brain regions important for somatosensation and contextual encoding of fear learning. **A** Schematic of brain regions involved in somatosensation. **B** Average of regions involved in somatosensation. A repeated measures ANOVA in these regions indicated a difference based on apoE isoform (*p* = 0.031, *F*(2,26) = 4.000), driven by the differences between E2 and E4 (*p* = 0.028). Z-scores of cFos levels were seen in (**C**) the intralaminar nucleus of the dorsal thalamus. **D** The thalamus, sensory-motor cortex related. **E** The posterior complex of the thalamus. **F** The parabrachial nucleus. **G** The locus coeruleus, and (**H**) the agranular insular area. Subsequent analysis identified apoE isoform-specific differences in the thalamus, sensory-motor cortex related (*p* = 0.048, *F*(2,26) = 3.417). The Bonferroni’s post hoc test revealed that the difference was driven by E2 vs. E3 mice (*p* = 0.048). **I** Schematic of brain regions involved in contextual encoding. **J** Average of regions involved in contextual encoding. A repeated measures ANOVA in these regions indicated a difference based on region (*p* = 0.040, *F*(3.494,90.837) = 2.743, Greenhouse-Geisser corrected) and apoE isoform (*p* = 0.002, *F*(2,26) = = 7.671). The Bonferroni’s post hoc test revealed an overall difference between E2 and E4 mice (*p* = 0.002) and a trend towards a difference between E3 and E4 mice (*p* = 0.078). Z-scores of cFos immunoreactivity levels were seen in (**K**) the piriform area. **L** The primary somatosensory area. **M** The subiculum, ventral part. **N** The perirhinal area. **O** The entorhinal cortex. **P** The hippocampus. Subsequent analysis identified apoE isoform-specific differences in the entorhinal cortex (*p* = 0.018, *F*(2,26) = 4.695) and the hippocampus (*p* = 0.045, *F*(2,26) = 3.506). Trends towards apoE isoform differences were seen in the primary somatosensory area (*p* = 0.074) and the perirhinal area (*p* = 0.074). The Bonferroni’s post hoc revealed that the difference in the entorhinal cortex was driven by E3 vs. E4 mice (*p* = 0.031), with a trend towards a difference between E2 vs. E4 mice (*p* = 0.057). Data are presented in minimum to maximum boxplots. Each point represents an individual animal. *p < 0.05.

There was a difference between isoforms in regions important for somatosensation (*p* = 0.031; Fig. 2A, B), driven by the difference between E2 and E4 mice (*p* = 0.028). The sensory-motor cortex-related area was different based on apoE isoform (*p* = 0.048, MANOVA; Fig. 2D), with *post hoc* testing indicating a trend towards a difference in E2 compared to E4 mice (*p* = 0.058). There was no difference in the posterior complex of the thalamus (Fig. 2E), intralaminar area (Fig. 2C), parabrachial nucleus (Fig. 2F), locus coeruleus (Fig. 2G), or agranular insular area (Fig. 2H).

Analysis of regions important for contextual encoding revealed an effect of region (*p* = 0.040, Greenhouse-Geisser corrected; Fig. 2I, J), indicating that these regions have distinct patterns of cFos activation. There was also an effect of isoform (*p* = 0.002). Again, E4 mice had lower cFos levels than E2 mice (*p* = 0.002) and trended towards lower levels than E3 mice (*p* = 0.078). There were no differences between E2 and E3 mice. Follow-up analyses on specific sub-regions revealed that cFos immunoreactivity was different in the entorhinal cortex (*p* = 0.018; Fig. 2O) and hippocampus (*p* = 0.045; Fig. 2P), though *post hoc* analysis only revealed a difference between E3 and E4 (*p* = 0.031) and a trend towards a difference between E2 and E4 (*p* = 0.057) in the entorhinal cortex. There was no difference in the primary somatosensory area (Fig. 2L), perirhinal area (Fig. 2N), piriform area (Fig. 2K) or subiculum, ventral part (Fig. 2M).

**Fig. 3:**
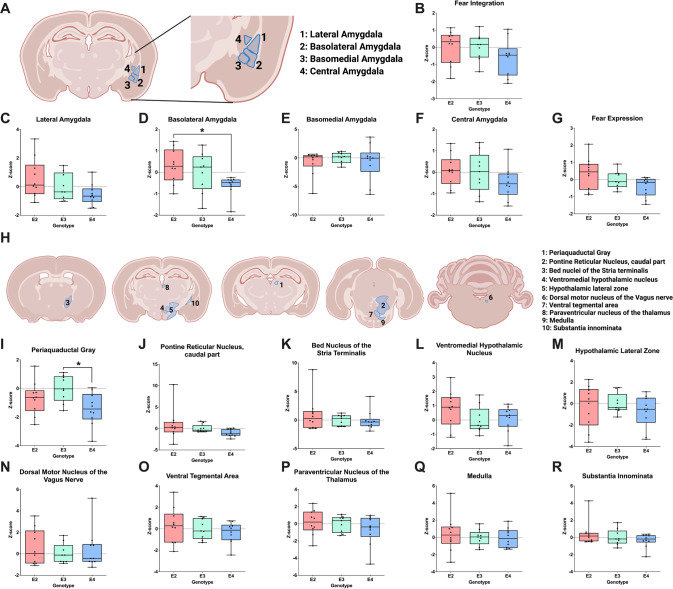
cFos immunoreactivity in brain regions important for integration of contextual fear learning and fear expression. **A** Schematic of brain regions involved in fear integration. **B** Average of regions involved in fear integration. A repeated measures ANOVA in these regions did not reveal any overall differences between apoE isoforms (*p* = 0.272). Z-scores of cFos immunoreactivity levels were seen in (**C**) the lateral amygdala, **D** The basolateral amygdala, **E** The basomedial amygdala, **F** The central amygdala. Subsequent analysis identified apoE isoform-specific differences in the basolateral amygdala (*p* = 0.035, F(2,26) = 3.810) and a trend towards apoE isoform differences in the lateral amygdala (*p* = 0.078). The Bonferroni’s post hoc test revealed that the difference in the basolateral amygdala was driven by E2 vs. E4 mice (*p* = 0.035). **G** Average of regions involved in fear expression. **H** Schematic of brain regions involved in fear expression. A repeated-measures ANOVA in these regions revealed a trend towards an overall differences between apoE isoforms (*p* = 0.055, *F*(2,26) = 3.239). Z-scores of cFos immunoreactivity levels were seen in (**I**) the periaqueductal gray. **J** The pontine reticular nucleus, caudal part. **K** The bed nucleus of the stria terminalis. **L** The ventromedial hypothalamic nucleus. **M** The hypothalamic lateral zone. **N** the dorsal motor nucleus of the vagus nerve. **O** The ventral tegmental area. **P** The paraventricular nucleus of the thalamus. **Q** The medulla and (**R**) the substantia innominata. Subsequent analysis identified apoE isoform-specific differences in the periaqueductal gray (*p* = 0.029, *F*(2,26) = 4.092). The Bonferroni’s post hoc test revealed that the difference was driven by E3 vs. E4 mice (*p* = 0.025). Data are presented in minimum to maximum boxplots. Each point represents an individual animal. *p < 0.05.

Next, we analyzed cFos immunoreactivity in the amygdalar nuclei (Fig. 3A). There were no overall isoform-dependent differences in cFos immunoreactivity (*p* = 0.272; Fig. 3B), but there was a difference in the basolateral amygdala (*p* = 0.035; Fig. 3D) with E2 mice having higher cFos levels than E4 mice (*p* = 0.035). There was no difference in the lateral (Fig. 3C), basomedial (Fig. 3E), or central amygdala (Fig. 3F).

Lastly, we explored apoE isoform differences in brain regions important for fear expression (Fig. 3F). There was a trend toward a main effect of apoE isoform (*p* = 0.055; Fig. 3G), again with E4 mice trending towards lower cFos levels than E2 mice (*p* = 0.052). There were apoE differences in the periaqueductal gray (*p* = 0.029). E4 mice had less cFos immunoreactivity than E3 mice (*p* = 0.025; Fig. 3I). No differences were found in the pontine reticular nucleus, caudal part (Fig. 3J), bed nuclei of the stria terminalis (Fig. 3K), ventromedial hypothalamic nucleus (Fig. 3L), hypothalamic lateral zone (Fig. 3M), dorsal motor nucleus of the vagus nerve (Fig. 3N), ventral tegmental area (Fig. 3O), paraventricular nucleus of the thalamus (Fig. 3P), medulla (Fig. 3Q), or substantia innominata (Fig. 3R).

Taken together, these analyses suggest that apoE isoform-dependent differences in contextual fear circuitry are localized to areas important for somatosensation and contextual encoding, with lower cFos immunoreactivity levels in E4 mice.

### ApoE isoform-dependent differences in co-activation of cFos immunoreactivity across regions important for contextual fear learning in young female mice

**Fig. 4:**
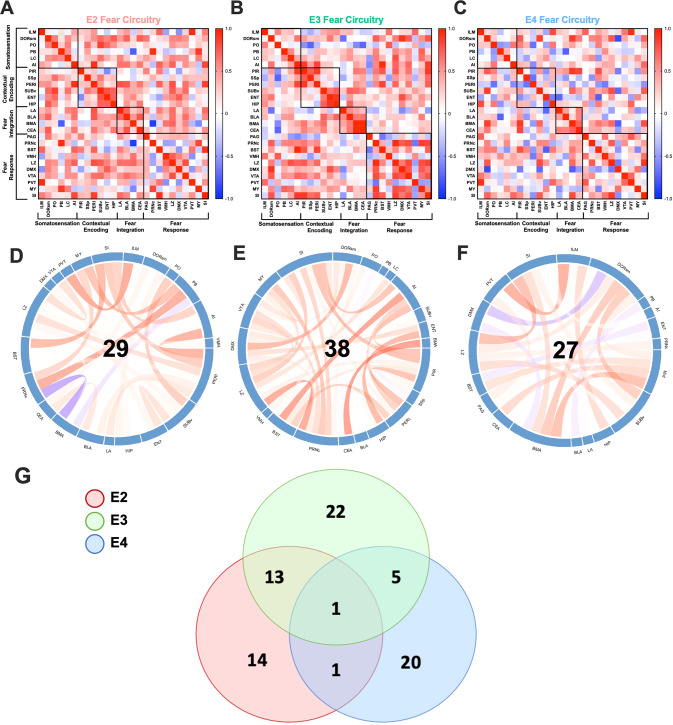
Correlation matrices of brain regions involved in contextual fear learning. Pearson’s correlations were run for (**A**) E2, (**B**) E3, and (**C**) E4 mice to identify co-activation of cFos immunoreactivity across regions. Comparison of the correlation matrices revealed that E4 mice were different from E3 mice (*p* = 0.0174, $\hat T$ = 3.9302). To identify the drivers of this difference, we mapped the regions that had *r* values > 0.7 or < −0.7 and generated chord diagrams for (**D**) E2, (**E**) E3, and (**F**) E4 animals. **G** A Venn diagram of the number of regions that were identified as having the strongest correlations in each of the genotypes, and their overlap.

Obtaining data from the entire brain allowed us to analyze the co-activation of cFos cells within the fear circuitry. We calculated the Pearson’s coefficient for these regions and built covariance matrices categorized into the 4 sub-categories for each apoE isoform (Fig. 4A–C), then analyzed these matrices. The co-activation of cFos immunoreactivity in E4 mice was different from that in E3 mice (*p* = 0.0174; Fig. 4B, C). There was no difference between E2 and E3 mice (*p* = 0.208; Fig. 4A, B) or E2 and E4 mice (*p* = 0.2348). These results suggest that communication between fear-related brain regions may be specifically altered in young female E4 mice.

To identify what correlations were driving these results, we examined all brain regions that had *r*-values above + 0.7 or below −0.7. Figure 4D–F show these analyses, which revealed a few major patterns between the apoE isoforms (Fig. 4G). First, there was only one overlap in regional correlations across the three isoforms (the agranular insular area and the substantia innominata). Second, E2 and E3 mice showed high correlations between contextual encoding regions, while E4 mice did not have any strong correlations between these regions. Third, E4 mice showed more strong negative correlations (5 total) than either E2 or E3 (2 each). Finally, E4 mice showed the least overlap with either E2 or E3.

### Age, sex, and apoE isoform affect hippocampal DSBs and IEG expression at baseline and after fear conditioning

To assess possible hippocampus-specific changes, we used immunoreactivity and ChIP-ddPCR to measure DSBs and IEG expression before (behaviorally naïve) and after fear conditioning.

**Fig. 5:**
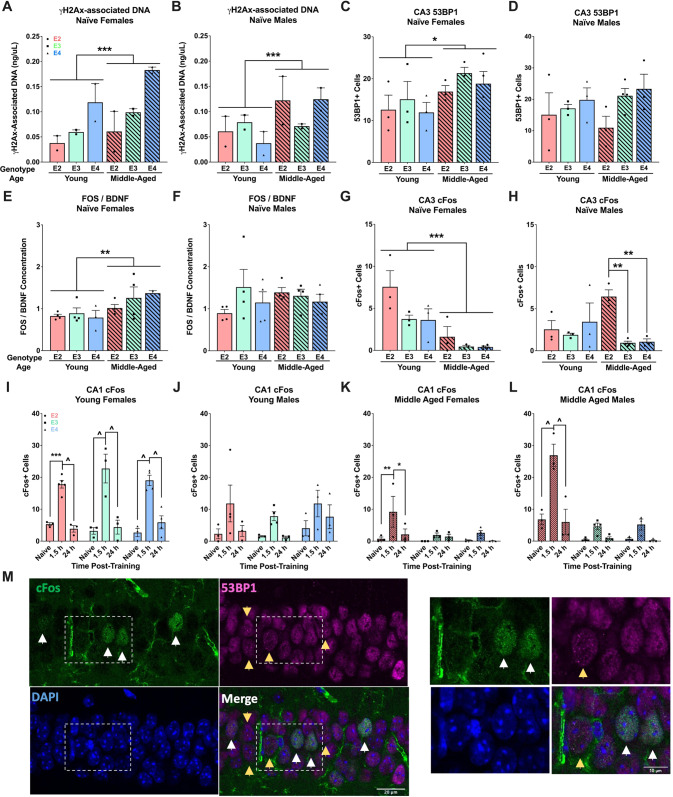
DSB and IEG expression in young and middle-aged male and female mice before or after fear training. **A** Amount of DNA pulled down by γH2Ax ChIP in naïve females. There was an effect of age (*p* < 0.001, *F*(1,18) = 14.501) and genotype (*p* < 0.001, *F*(2,18) = 29.522). E4 mice had more γH2Ax-associated DNA than E2 (*p* < 0.0001) or E3 (*p* < 0.0001). **B** Amount of DNA pulled down by γH2Ax ChIP in naive males. There was an effect of age (*p* = 0.002, *F*(1,18) = 13.321) and an age-by-genotype interaction (*p* = 0.021, *F*(2,18) = 4.847). **C** Number of cells with 53BP1 foci in the CA3 of naïve females. Middle-aged animals had more 53BP1 than young animals (*p* = 0.029, *F*(1, 13) = 6.036). **D** Number of cells with 53BP1 foci in the CA3 of naïve males. There were no effects. **E** Relative Fos expression in the hippocampus of naïve females. Middle aged animals had more than young animals (*p* = 0.008, *F*(1,16) = 9.082). **F** Relative Fos expression in the hippocampus of naïve males. There were no effects. **G** Number of cells with 53BP1 foci in the CA3 of naïve females. There was an effect of age (*p* = 0.0003, *F*(1,13) = 24.16) and genotype (*p* = 0.045, *F*(2,13) = 3.975). **H** Number of cells with 53BP1 foci in the CA3 of naive males. There was an effect of genotype (*p* = 0.025, *F*(2, 13) = 4.941) and an age-by-genotype interaction (*p* = 0.023, *F*(2,13) = 5.096). Middle-aged E2 had more than middle-aged E3 (*p* = 0.004) and E4 (*p* = 0.007) mice. **I** cFos in the CA1 of young females. There was an effect of time post-training (*p* < 0.0001, *F*(2, 22) = 67.61); mice euthanized at 1.5 h had mor cFos than in behaviorally naïve or 24 h for all genotypes. **J** cFos in the CA1 of young males. There was an effect of time post-training (*p* = 0.015, *F*(2,20) = 5.233); post hoc testing did not reach significance. **K** cFos in the CA1 of middle-aged females. There was an effect of time post-training (*p* = 0.011, *F*(2,20) = 5.656) and a trend towards an effect of genotype (*p* = 0.063). Only E2 mice euthanized at 1.5 h had higher cFos than behaviorally naïve or 24 h. **L** cFos in the CA1 of middle-aged males. There was an effect of time post-training (*p* < 0.0001, *F*(2,21) = 29.38) and genotype (*p* < 0.0001, *F*(2,21) = 37.70) and a time-by-genotype interaction (*p* < 0.001, *F*(4,21) = 8.85). Only E2 mice euthanized at 1.5 h had higher cFos than behaviorally naive or 24 h. **M** Representative images of the CA1. White arrows indicate cFos+ cells, yellow arrows indicate 53BP1 + cells. *Right*: zoomed images of the white boxes. Data are presented as the mean ± SEM. *p < 0.05, **p < 0.01, ***p < 0.001, ^*p* < 0.0001.

In behaviorally naïve females, there were differences in the amount of DNA pulled down by γH2Ax based on age (*p* < 0.001) and isoform (*p* < 0.001): middle-aged females had more γH2Ax-bound DNA than young females, and E4 more than E2 (*p* < 0.001) and E3 (*p* < 0.001; Fig. 5A) mice. Behaviorally naïve males similarly showed a difference based on age (*p* = 0.002) and an age by isoform interaction (*p* = 0.021). Middle-aged males also had higher levels than young males (Fig. 5B). Counts of the number of cells that contained 53BP1 foci in the CA3 also revealed a main effect of age (*p* = 0.028) in females (Fig. 5C). This was not seen in males (Fig. 5D). There was no effect of isoform and there were no effects in fear conditioned animals (Supplementary Tables 2 and 3). Together, these data indicate an increase in baseline DSBs with age that are partially dependent on sex and apoE isoform.

We next assessed baseline levels of IEG expression. Analysis of Fos levels normalized to BDNF (a control marker) again revealed a main effect of age (*p* = 0.008) in females (Fig. 5E) but not males (Fig. 5F). There was no effect of genotype, nor any differences in fear conditioned animals. There were no differences in Npas4 levels (Supplemental Table 2). There was a main effect of age in the number cFos+ cells in the CA3 region of females (*p* < 0.001; Fig. 5G). In contrast, there was a difference in cFos+ cells in behaviorally naive males based on isoform (*p* = 0.025) and there was an age by isoform interaction (*p* = 0.023; Fig. 5H). Middle-aged E2 males had more cFos+ cells than E3 (*p* = 0.004) and E4 (*p* = 0.007) males. These data point to a change in baseline IEG expression with aging that is dependent on sex and apoE isoform.

Subsequently, we analyzed cFos immunoreactivity in the CA1 and CA3 regions in three conditions (behaviorally naive, 1.5 h, or 24 h post-fear learning). Compared to behaviorally naïve and 24 h post-fear learning, the number of cFos+ cells in the CA1 region of young, female mice was higher at the 1.5 h time point in all isoforms (*p* < 0.0001; Fig. 5I). This effect was also seen in young, male mice (*p* = 0.015), though *post hoc* testing did not reveal significance between conditions within isoforms (Fig. 5J). The effect of condition was observed in middle-aged females (*p* = 0.011; Fig. 5K) and middle-aged males (*p* < 0.0001; Fig. 5L); in both sexes, E2 mice were the only to show increases in cFos+ cells at 1.5 h compared to behaviorally naïve and 24 h post-learning. Middle-aged males also showed an effect of genotype (*p* < 0.0001) and a time-by-genotype interaction (*p* < 0.001). Similar sex- and isoform-dependent patterns were observed in the CA3 region (Supplemental Fig. 1A–E). Representative CA1 images are shown in Fig. 5M.

## Discussion

Our results provide insight into alterations to IEG signaling dependent on age, sex, and human apoE isoforms. Young E4 female mice displayed lower cFos immunoreactivity levels across regions important for somatosensation and contextual encoding, including the thalamus, sensory-motor cortex-related; entorhinal cortex (ETX); hippocampus; basolateral amygdala; and periaqueductal gray. E4 mice also showed increased time freezing during ISIs and altered co-activation of brain regions, with more negative correlations across regions than E3 mice. There was an increase in hippocampal DSB markers in middle-aged animals and female E4 mice, as well as age-, sex-, and apoE-dependent alterations to baseline and post-fear conditioning Fos levels. These changes in IEG expression may play a role in the apoE isoform-specific risks for neurological disorders.

The observed apoE differences in time freezing during the ISIs are in line with our earlier studies [42, 61]. E2 mice show impaired fear extinction compared to E3 and E4 mice [11, 41]. In humans, the E2 isoform has been relatively less-studied due to lower ε2 allele frequency (8%) in the general population [6]. Heterozygous E2-carriers show a decreased risk to develop LOAD and increased longevity [62–65], though E2 is associated with other neurological diseases, including higher rates of PTSD, PTSD symptom severity as measured by the Clinician-Administered PTSD-scale, and alcohol use disorders [10, 11, 66]. Impaired fear extinction in mice closely matches one of the primary symptoms of PTSD—the persistence of traumatic memories. As there is a lack of data on fear acquisition related to apoE isoform, our results add to knowledge about distinctions during a highly salient learning event.

Compared to E3, E4 has also been linked to PTSD. E4-homozygous combat veterans with high levels of combat exposure have a higher lifetime prevalence of PTSD and increased PTSD severity as measured by the Davidson Trauma Scale [67], a self-report survey [68, 69]. While highly reliable [70], the different rating scales used could partially account for the distinct apoE isoform-related results. Moreover, there is no increased risk for PTSD in E4-carriers with low combat exposure. The neuropathological impact of traumatic brain injury (TBI) is shown to be worse in E4 carrying people [71, 72] and mice [73–75]. This includes increased tau phosphorylation, accumulation of amyloid β (Aβ), and inflammation. Thus, the interaction between combat exposure (i.e., repeated TBIs) with apoE isoform may contribute to the divergent PTSD diagnoses and presentations. PTSD also might have a bi-directional relationship with dementia: PTSD increases the risk for dementia, and dementia increases the risk for delayed-onset PTSD [76]. ApoE isoform was not assessed in that study. The interactions between life experience, sex, and apoE isoform on PTSD and AD development are complex; our results that middle-aged E2 mice maintain high levels of post-training cFos activation (while E3 and E4 fail to do so) add clarity to possible underlying neural mechanisms.

Dysregulation in IEGs has been implicated in many neurological diseases, including increased circulating Egr1 in schizophrenia [77, 78], increased Arc in the amygdala in major depressive disorder [79, 80], and increased cFos and cJun in the hippocampus and amygdala in AD [81–83]. Rodent research has highlighted dysregulation of IEGs in AD and PTSD models. Young (4–6-month-old) mice carrying amyloid precursor protein (APP) mutations—an autosomal dominant cause of AD—have increased cognitive impairments and hippocampal delta-FosB levels, leading to downregulation of cFos [84]. APP knock out mice display high baseline levels of Egr1, cFos, and BDNF in the prefrontal cortex [85], which might interfere with the expected IEG increase following novelty, suggesting that APP is important for proper IEG regulation. PTSD models show elevated cFos immunoreactivity in the bed nucleus of the stria terminalis, paraventricular hypothalamus, paraventricular thalamus, basolateral amygdala (BLA), central amygdala, medial amygdala, and periaqueductal gray (PAG) [86, 87]. The BLA and PAG were two regions we identified with apoE-dependent differences, with E4 mice having less cFos immunoreactivity. It is possible that the higher cFos levels in E2 and/or lower levels in E4 mice could contribute to different fear expression—namely, E4 mice freezing more during training and E2 mice exhibiting impaired extinction [11].

Upstream, apoE influences APP transcription via phosphorylation of cFos [30]. In male-derived iPSCs, E4 stimulates phosphorylation of ERK1 and cFos, increases APP levels, and increases Aβ secretion more than E3, and E3 does so more than E2 [30]. RNAi-mediated inhibition of cFos decreases APP levels [30]. There is also a stepwise decrease from E4 to E3 to E2 in activation of synapse-building genes via CREB phosphorylation [88]. These might appear paradoxical to our current findings, however the iPSC experiments involved exogenous apoE in a reduced system. E4 carriers have lower apoE levels than non-E4 carriers throughout the body independently of AD diagnosis [89–91]. E4 mice on a high fat diet (HFD) did not show increases in cFos and Arc in the hippocampus, cortex, or hypothalamus, while E3 mice on a HFD did [29]. Thus, it is conceivable that lower apoE levels in E4 carriers lead to less IEG expression. Remarkably, there was higher freezing in E4 mice despite generally lower cFos expression, though alterations in brain activation have been reported in E4 carriers in the absence of cognitive differences [14, 20]. Additionally, young adult E2 carriers had larger hippocampal gray matter and relied more on hippocampus-dependent strategies to solve a navigation task [92]. We found apoE-dependent differences in hippocampal cFos+ cells in young and middle-aged mice, where E2 mice generally had more hippocampal cFos immunoreactivity. Our results suggest E2-specific increased hippocampal activation during fear learning, complementing the human findings.

The data from human imaging studies are in line with the distinctions we observed when we analyzed regional correlations. There was a difference between the correlation matrices of E3 and E4 animals only. When we examined the strongest correlations (*r* > 0.7 or *r* < −0.7), there was a single overlapping correlation between all 3 isoforms. This finding—that the 3 isoforms are more separate than similar for this measure—might relate to the differential risks associated with E2 and E4. Moreover, E4 mice did not show any strong positive correlations within the contextual encoding regions and had more strong negative correlations than E2 or E3 mice. This was clear when looking at the ETX and hippocampus correlation: E4 mice displayed a negative correlation (−0.621), while E2 and E3 mice displayed positive correlations (0.71 and 0.906, respectively). Analysis of functional connectivity involving the ETX in an older human population reported similar results: E2 carriers showed the strongest functional connectivity of the ETX with the frontal and middle temporal gyrus; E3 carrier with the postcentral gyrus, inferior parietal lobule, and insula (among others); and E4 carriers with the precuneus, angular gyrus, superior parietal lobule, and posterior cingulate (among others) [93]. This population included healthy controls and patients with MCI and showed that E4 alterations in functional connectivity were associated with increased speed of dementia onset, while the alterations in E2 carriers were associated with protection.

In humans, there are increases in neuronal γH2Ax and 53BP1 in brains from patients with MCI or AD compared to healthy, age-matched controls [33]. Increased oxidative stress, which induces DSBs, is seen in typical and diseased aging [94–98]. Hippocampal 8-Hydroxydeoxyguanosine is higher, while 53BP1 and PTEN (another DNA repair protein) are decreased in post-mortem tissue from diagnosed AD patients, suggesting impaired DSB repair [95]. Age is also associated with increased DNA damage in the frontal cortex, leading to down-regulation of genes important for synaptic plasticity [96]. Decreased DNA repair—specifically in accurate 53BP1 recruitment—was also observed in aged human cell cultures [94–98]. Here, we found an increase in hippocampal γH2Ax and 53BP1 levels in middle-aged animals and an increase in relative Fos immunoreactivity, indicative of increased aberrant signaling with aging. This result was more pronounced in E4 females, paralleling others’ findings that age, sex, and E4 synergistically increase risk for AD [99].

We did not observe increases in DSBs post-training, likely due to differences in γH2Ax and 53BP1 function and kinetics, nor increases in DSB-associated IEGs after fear conditioning as reported by others [52, 100]. Both Navabpour et al. [52] and Stott et al. [100] observed fear conditioning-induced increases in *Npas4*, but not *Fos*. This discrepancy with our study could be due to differences in methodology and/or mechanisms of distinct IEGs. However, there was an increase in γH2Ax-associated IEG expression at baseline in middle-aged animals, and in E4 females. These results mirror the previously reported data showing that baseline levels of γH2Ax are higher in APP mutant mice [32]. Notably, that study involved 4–7-month-old mice and collapsed sexes for analysis, while we assessed young (3 months) and middle-aged (12 months) mice without collapsing sexes. Another study reported increased hippocampal γH2Ax, but not 53BP1 levels, in both young (5 months) and aged (16 months) APP/PS1 mice, in line with our results [101].

Altogether, these data highlight that apoE and sex interact and have distinct age-dependent effects on DSBs and IEG expression. These effects, specifically in E4 carriers, could be a driving factor in enhanced E4-specific risk for neurological diseases.

### Supplementary information

supplemental information for manuscript

## Data Availability

All data is freely available upon request.
