# Supplementary material for: Age, sex, and apolipoprotein E isoform alter contextual fear learning, neuronal activation, and baseline DNA damage in the hippocampus
Source: Mol Psychiatry. 2023 Feb 2;28(8):3343–54. doi: 10.1038/s41380-023-01966-8 (PMC10618101; doi:10.1038/s41380-023-01966-8)
Supplement: Supplementary file 1 — supplemental information for manuscript [file 41380_2023_1966_MOESM1_ESM.docx]

**Supplemental Material**

***Title:*** Age, sex, and apolipoprotein E isoform alter contextual fear learning, neuronal activation, and baseline DNA damage in the hippocampus

Methods:

*Contextual Fear Conditioning*

Mice were placed into plexiglass chambers with a shock grid (Med Associates, St. Albans, Vermont) and allowed to explore for a baseline period of 148 s. A 2 s, 0.5 mA shock was administered, followed by a 148 s inter-stimulus interval (ISI), a second 2 s shock, and a final 60 s ISI. After these 6 minutes, mice were removed from the fear conditioning chambers. Chambers were thoroughly cleaned with 0.5% acetic acid after each trial. Trials were recorded with Video Freeze software (Med Associates). Time freezing, defined as the cessation of all movement except breathing, and average indices of motion, measured by changes in video-pixel composition in arbitrary units (au), were analyzed.

*Tissue collection*

Experiment 1: Two hours after fear conditioning, mice were injected with a 50 mg/ml lethal ketamine-xylazine cocktail to capture peak cFos levels [49, 50]. Mice were intracardially perfused with 15 ml of ice-cold saline followed by 15 ml of 4% PFA. Brains were removed and stored overnight in 4% paraformaldehyde (PFA), then switched to phosphate-buffered saline (PBS) 24 h later. The brains were sent to Certerra, Inc. (acquired by Certego Therapeutics) for whole-brain clearing, cFos staining, and imaging as previously described [43]. Certerra’s standard protocol includes incubating hemispheres in a rabbit-anti-cFos monoclonal antibody (1:450, Cell Signaling #2250S) for 1 week followed by incubation in a donkey-anti-rabbit AlexaFluor647 antibody (1:400, Fisher, #A31573) for 1 week.

Experiment 2: Mice were perfused as described above. Brains were switched to cryopreserve after 24 h. Mice in the fear training group were trained in contextual fear conditioning then returned to their home cages and euthanized 1.5 h later. This time point was chosen to find the peak overlap between cFos expression and 53BP1 [50, 51]. Mice in the fear training + recovery group were trained in the same contextual fear conditioning, then returned to their home cages and perfused 24 h later. Behaviorally naïve mice were never exposed to fear conditioning.

Experiment 3: Mice in the fear training group were trained in contextual fear conditioning; 5 min after completing the trial, they were euthanized by rapid cervical dislocation and decapitation. This time point was chosen based on data indicating that γH2Ax-DNA interactions are highest within 5 min after stimulation [52]. Behaviorally naïve mice were never exposed to fear conditioning. The hippocampus and cortex were dissected and flash frozen in liquid nitrogen, then stored at -80°C until use.

*Dual-label immunofluorescence & microscopy*

Free-floating brain sections cut to 40 µm thickness were rinsed in 1x PBS and blocked in 4% normal goat serum (NGS) before overnight incubation in rabbit-anti-53BP1 (1:3000, Bethyl Labs, # A300-272A). On day 2, sections were rinsed and incubated in goat-anti-rabbit antibody (AlexaFluor647, Invitrogen, # A-21245, 1:250) for 2.5 h. Sections were thoroughly rinsed in 1x PBS, blocked again in 4% NGS, and incubated in mouse-anti-c-Fos overnight (1:500, PhosphoSolutions, # 309-cFOS). On day 3, sections were rinsed an incubated in goat-anti-mouse antibody (AlexaFluor488, Invitrogen, #A-11001, 1:250) for 2.5 h, then washed in 1x PBS. Following washes, 2.5 µg/mL DAPI (Sigma D9542) in PBS was applied for 20 min. Sections were rinsed, then slide mounted with CitiFluor CFMR2 Antifadent Solution and sealed with Biotium CoverGrip Coverslip Sealant.

Three sections were imaged per animal. Z-stack images at 0.25 µm steps were taken using a Zeiss LSM 980 with Airyscan 2 at 63x zoom. Images were taken of the CA3 and CA1. The number of cells with 53BP1 foci, cFos, and co-localized signal were manually counted using ImageJ (NIH, Bethesda, MD).

*Chromatin Immunoprecipitation & digital droplet PCR*

Hippocampal tissue was chopped finely in ice-cold PBS, homogenized using 18g followed by 21g needles, fixed with 1% formaldehyde for 10 min at room temperature, quenched with glycine for 5 min at room temperature, washed in cold PBS 3 x 10 min, incubated in MAGnify lysis buffer with proteinase inhibitor cocktail for 5 min, and sonicated for 5 min using a Covaris s220 sonicator. Samples were then spun down to remove cell debris. 1% of the sample was taken for chromatin input, while the rest was incubated with 2 µg of γH2Ax antibody (Cell Signal, #9718) overnight at 4°C. DNA reverse-crosslinking and purification were done according to the MAGnify protocol. Concentrations for ChIP and input samples were measured using the Qubit dsDNA High Sensitivity kit (ThermoFisher). Samples were stored at -20°C until use in digital droplet PCR (ddPCR).

Digital droplet PCR (ddPCR) was used to assess cFos, Npas4, and BDNF expression [56]. Samples were prepared according to the BioRad QX200™ Droplet Digital™ PCR System manual (BioRad, Hercules, CA). Briefly, 10 µL of ChIP sample were prepared per 22 µL reaction with EvaGreen QX200 ddPCR SuperMix and BioRad primers designed for Fos (#dMmuEG5059391), Npas4 (#dMmuEG5061441), or BDNF (#dMmuEG5062297). Samples were converted into droplets with the QX200™ system, then PCR done using a T1000 thermal cycler (BioRad) with the following protocol: 95°C for 5 min, then 34 cycles of 96°C for 30 s then 58°C for 1 min, stabilization at 4°C for 5 min, then 90°C for 5 min. Fluorescence was measured on a QX200™ droplet reader on the EvaGreen channel and analyzed using QuantaSoft™ software. The number of positive droplets normalized to DNA input and copies/ng DNA were analyzed.

*Statistical analysis*

For fear conditioning, average motion (arbitrary units) and percent time freezing were analyzed. We used an ANOVA for average motion during the baseline period and average motion in response to the shocks. For learning, we analyzed the percent time spent freezing during the ISIs with a repeated-measures ANOVA.

The number of cFos+ cells, 53BP1+ cells, and cFos-53BP1+ cells in the CA1 and CA3 were analyzed with a multi-way ANOVA including time post-training and genotype as between group variables. The concentration of cFos, Npas4, and BDNF (measured in copies/µL) was normalized to total DNA and analyzed with an ANOVA with genotype, age, and time post-training as between group variables. Additionally, to determine relative enrichment, cFos and NPAS4 concentrations were compared to BDNF levels.

For whole-brain analysis, ClearMap was applied to collected light-sheet images to segment brain regions and count number of cFos+ cells [57]. We first analyzed the raw number of cFos+ cells throughout the brain. We discovered significant differences between cohorts, likely due to the sensitive nature of IEGs. As such, we transformed the cFos counts into Z-scores, normalized to the E3 animals in each cohort.

We then proceeded to analyze differences in cFos activation across the brain using the Z-scores. We first identified brain regions known to be important for contextual fear learning [34, 35] and organized them into 4 distinct subcategories: somatosensation, contextual encoding, fear integration, and fear expression. (**Supplemental table 1**). identifies these regions, the acronyms, and their primary function related to contextual fear learning. We then used repeated-measures ANOVAs in these sub-categories to identify regional and isoform-dependent differences, with Bonferroni’s post hoc corrections to compare the isoforms. To localize regional differences, we subsequently used MANOVAs with Bonferroni corrections to identify isoform differences in specific brain regions.

We have previously used this type of data as an indirect measure of connectivity [44, 45]. Here, we calculated the Pearson’s coefficient for all fear-related brain regions. We then built correlation matrices and compared the matrices to identify apoE isoform-specific changes in co-activation of cFos, as previously described using a method previous described [58]. The statistic defined by Chang and colleagues [58] tests whether two covariance matrices are identical. Critically, this method is sensitive to small numbers of pairwise differences in covariance matrices by testing whether the maximum element-wise difference between the two covariance matrices is 0, making the test appropriate for examining differences in covariance matrices in cases where small numbers of pairwise differences may drive overall differences in covariance patterns, such as in gene expression [59] or neuronal connectivity [45]. The test statistic, $\hat{T}$, is defined as the maximum of the squared, element-wise differences between the two covariance matrices, normalized by its variance. The *p*-value is determined by creating an empirical null distribution of $\hat{T}$ using a multiplier bootstrap [60] and counting the proportion of bootstrap statistics that are larger than the test statistic. This was shown to be appropriate even in cases where the number of dimensions is greater than the number of subjects [58].

**Supplemental Table 1**. List of brain regions involved in contextual fear learning.

| ROIs | Full Name | Functional Classification | Detailed Function |
| --- | --- | --- | --- |
| ILM | Intralaminar area of the dorsal thalamus | Somatosensation | Somatic sensation |
| DORsm | Thalamus, sensory-motor cortex related | Somatosensation | Sensation of aversive unconditioned stimulus |
| PO | Posterior complex of the thalamus | Somatosensation | Sensation of aversive unconditioned stimulus |
| PB | Parabrachial Nucleus | Somatosensation | Sensation of aversive unconditioned stimulus |
| LC | Locus coeruleus | Somatosensation | Sensation of aversive unconditioned stimulus |
| AI | Agranular insular area | Somatosensation | Somatic sensation |
| PIR | Piriform area | Contextual Encoding | Olfaction |
| SSp | Primary somatosensory area | Contextual Encoding | Integration of sensation of aversive unconditioned stimulus |
| PERI | Perirhinal cortex | Contextual Encoding | Temporal encoding |
| SUBv | Subiculum, ventral part | Contextual Encoding | Contextual encoding |
| ENT | Entorhinal cortex | Contextual Encoding | Contextual encoding |
| HIP | Hippocampus | Contextual Encoding | Contextual encoding |
| LA | Lateral Amygdala | Fear Integration | Integration of sensation of aversive unconditioned stimulus |
| BLA | Basolateral amygdala | Fear Integration | Integration of associative learning |
| BMA | Basomedial amygdala | Fear Integration | Integration of associative learning |
| CEA | Central amygdala | Fear Integration | Output of autonomic components of emotion |
| PAG | Periaqueductal gray | Fear Expression | Freezing |
| PRNc | Pontine reticular nucleus, caudal part | Fear Expression | Potentiated startle |
| BST | Bed nuclei of the stria terminalis | Fear Expression | Corticosterone release |
| VMH | Ventromedial hypothalamic nucleus | Fear Expression | Corticosterone release |
| LZ | Hypothalamic lateral zone | Fear Expression | Cardiovascular response |
| DMX | Dorsal motor nucleus of the vagus nerve | Fear Expression | Cardiovascular response |
| VTA | Ventral tegmental area | Fear Expression | Corticosterone release |
| PVT | Paraventricular nucleus of the thalamus | Fear Expression | Corticosterone release |
| MY | Medulla | Fear Expression | Cardiovascular response |
| SI | Substantia innominate | Fear Expression | Arousal |

^1^ Brain regions were organized into 4 functional classifications for analysis purposes. The order listed in the table corresponds to the order presented in the heat maps in Figure 4A-C.

**Supplemental Table 2.** Amount of DNA and PCR product following ChIP-ddPCR^1^.

| Genotype | Sex | Age | Behavioral Group | DNA Input | γH2Ax-bound DNA | Fos Concentration | Npas4 Concentration | BDNF Concentration |
| --- | --- | --- | --- | --- | --- | --- | --- | --- |
| E2 | Females | Young | Naïve | 0.916 ± 0.164 | 0.038 ± 0.01 | 173.55 ± 51.53 | 251.88 ± 86.88 | 209.24 ± 60.05 |
|  |  |  | Fear Conditioned | 1.072 ± 0.468 | 0.076 ± 0.04 | 75.52 ± 6.14 | 132.90 ± 14.95 | 106.95 ± 23.19 |
|  |  | Middle-Aged | Naïve | 1.275 ± 0.235 | 0.061 ± 0.04 | 209.71 ± 73.58 | 333.40 ± 105.07 | 223.49 ± 86.13 |
|  |  |  | Fear Conditioned | 1.134 ± 0.153 | 0.088 ± 0.03 | 91.50 ± 16.33 | 182.14 ± 22.03 | 131.99 ± 32.36 |
|  | Males | Young | Naïve | 1.144 ± 0.296 | 0.061 ± 0.03 | 144.00 ± 10.56 | 262.84 ± 31.98 | 162.47 ± 5.75 |
|  |  |  | Fear Conditioned | 1.387 ± 0.433 | 0.141 ± 0.03 | 112.84 ± 16.33 | 188.86 ± 37.96 | 136.36 ± 21.20 |
|  |  | Middle-Aged | Naïve | 1.25 ± 0.06 | 0.122 ± 0.05 | 130.35 ± 9.84 | 185.93 ± 9.24 | 96.15 ± 11.64 |
|  |  |  | Fear Conditioned | 1.075 ± 0.005 | 0.066 ± 0.001 | 67.82 ± 35.00 | 218.35 ± 15.15 | 82.70 ± 10.67 |
| E3 | Females | Young | Naïve | 1.014 ± 0.266 | 0.060 ± 0.004 | 157.58 ± 58.62 | 234.14 ± 66.56 | 199.88 ± 83.91 |
|  |  |  | Fear Conditioned | 1.187 ± 0.543 | 0.071 ± 0.04 | 113.97 ± 27.53 | 168.42 ± 41.55 | 122.16 ± 41.26 |
|  |  | Middle-Aged | Naïve | 1.885 ± 0.135 | 0.099 ± 0.01 | 170.77 ± 14.35 | 244.70 ± 42.20 | 140.10 ± 8.44 |
|  |  |  | Fear Conditioned | 1.215 ± 0.185 | 0.077 ± 0.02 | 92.74 ± 13.39 | 157.04 ± 24.33 | 134.93 ± 2.75 |
|  | Males | Young | Naïve | 0.904 ± 0.096 | 0.079 ± 0.01 | 154.25 ± 54.99 | 175.73 ± 22.13 | 106.12 ± 5.74 |
|  |  |  | Fear Conditioned | 1.300 ± 0.260 | 0.065 ± 0.02 | 117.98 ± 1.31 | 187.51 ± 20.08 | 123.72 ± 13.70 |
|  |  | Middle-Aged | Naïve | 1.405 ± 0.355 | 0.071 ± 0.01 | 110.05 ± 13.31 | 183.84 ± 15.02 | 84.62 ± 3.76 |
|  |  |  | Fear Conditioned | 1.063 ± 0.097 | 0.084 ± 0.05 | 138.43 ± 45.09 | 259.91 ± 62.41 | 223.63 ± 72.10 |
| E4 | Females | Young | Naïve | 1.463 ± 0.365 | 0.106 ± 0.04 | 84.81 ± 19.13 | 158.26 ± 31.47 | 107.19 ± 9.06 |
|  |  |  | Fear Conditioned | 0.968 ± 0.042 | 0.078 ± 0.01 | 70.99 ± 4.41 | 133.39 ± 2.97 | 93.44 ± 7.98 |
|  |  | Middle-Aged | Naïve | 1.55 ± 0.100 | 0.183 ± 0.01 | 110.91 ± 18.92 | 143.11 ± 12.24 | 104.99 ± 3.02 |
|  |  |  | Fear Conditioned | 1.155 ± 0.005 | 0.061 ± 0.01 | 72.31 ± 9.04 | 118.10 ± 24.12 | 111.80 ± 10.04 |
|  | Males | Young | Naïve | 1.36 ± 0.070 | 0.037 ± 0.02 | 226.21 ± 50.28 | 323.87 ± 25.23 | 212.94 ± 24.16 |
|  |  |  | Fear Conditioned | 1.74 ± 0.020 | 0.052 ± 0.03 | 218.83 ± 115.53 | 362.49 ± 127.00 | 252.98 ± 82.76 |
|  |  | Middle-Aged | Naïve | 1.675 ± 0.045 | 0.125 ± 0.02 | 134.45 ± 15.50 | 206.38 ± 19.97 | 117.30 ± 6.67 |
|  |  |  | Fear Conditioned | 1.279 ± 0.351 | 0.090 ± 0.01 | 130.45 ± 9.13 | 233.57 ± 21.49 | 172.26 ± 5.28 |

^1^ All data are measured in ng/µL and presented as averages ± SEM.

**Supplemental Table 3**. Number of cells containing 53BP1 foci and number of cells with colocalized cFos and 53BP1 foci^1^.

| Genotype | Sex | Age | Behavioral Group | 53BP1+ Cells CA1 | 53BP1+ Cells CA3 | cFos+53BP1 Colocalization CA1 | cFos+53BP1 Colocalization CA3 |
| --- | --- | --- | --- | --- | --- | --- | --- |
| E2 | Females | Young | Naïve | 28.1 ± 3.6 | See Fig. 5 | 2.4 ± 0.7 | 2.1 ± 0.4 |
|  |  |  | 1.5 h | 29.0 ± 2.3 | 18.1 ± 2.4 | 6.1 ± 0.8 | 3.0 ± 0.3 |
|  |  |  | 24 h | 24.0 ± 2.6 | 10.1 ± 2.0 | 1.9 ± 0.5 | 0.8 ± 0.3 |
|  |  | Middle-Aged | Naïve | 32.0 ± 4.1 | See Fig. 5 | 0.6 ± 0.3 | 0.6 ± 0.3 |
|  |  |  | 1.5 h | 29.2 ± 1.2 | 16.4 ± 2.5 | 2.9 ± 1.1 | 0.7 ± 0.4 |
|  |  |  | 24 h | 26.9 ± 3.7 | 22.4 ± 4.7 | 0.9 ± 0.4 | 0.8 ± 0.4 |
|  | Males | Young | Naïve | 31.3 ± 5.4 | See Fig. 5 | 0.9 ± 0.3 | 0.5 ± 0.1 |
|  |  |  | 1.5 h | 25.0 ± 2.6 | 14.8 ± 1.7 | 3.0 ± 0.4 | 2.0 ± 0.5 |
|  |  |  | 24 h | 32.6 ± 2.5 | 18.1 ± 1.6 | 1.4 ± 0.3 | 0.8 ± 0.5 |
|  |  | Middle-Aged | Naïve | 23.8 ± 3.0 | See Fig. 5 | 3.0 ± 1.2 | 1.0 ± 0.4 |
|  |  |  | 1.5 h | 37.3 ± 6.4 | 12.6 ± 1.7 | 11.4 ± 3.1 | 2.4 ± 0.3 |
|  |  |  | 24 h | 20.9 ± 6.3 | 21.6 ± 6.6 | 2.9 ± 1.3 | 1.8 ± 0.7 |
| E3 | Females | Young | Naïve | 21.5 ± 1.7 | See Fig. 5 | 0.9 ± 0.4 | 0.6 ± 0.2 |
|  |  |  | 1.5 h | 36.1 ± 3.4 | 13.9 ± 2.9 | 9.3 ± 1.3 | 2.9 ± 0.9 |
|  |  |  | 24 h | 29.4 ± 2.2 | 14.6 ± 1.1 | 1.9 ± 0.5 | 1.7 ± 0.5 |
|  |  | Middle-Aged | Naïve | 28.8 ± 2.1 | See Fig. 5 | 0.0 | 0.2 ± 0.1 |
|  |  |  | 1.5 h | 28.1 ± 2.5 | 23.8 ± 4.3 | 0.6 ± 0.1 | 0.4 ± 0.2 |
|  |  |  | 24 h | 30.2 ± 1.7 | 25.0 ± 2.7 | 0.3 ± 0.1 | 0.4 ± 0.2 |
|  | Males | Young | Naïve | 37.1 ± 3.4 | See Fig. 5 | 1.1 ± 0.2 | 0.9 ± 0.2 |
|  |  |  | 1.5 h | 38.5 ± 2.0 | 21.1 ± 1.6 | 5.7 ± 0.9 | 2.9 ± 0.4 |
|  |  |  | 24 h | 27.1 ± 3.3 | 17.6 ± 1.4 | 0.7 ± 0.2 | 1.3 ± 0.3 |
|  |  | Middle-Aged | Naïve | 30.0 ± 3.0 | See Fig. 5 | 0.3 ± 0.2 | 0.4 ± 0.1 |
|  |  |  | 1.5 h | 34.6 ± 3.4 | 24.5 ± 0.7 | 2.3 ± 0.8 | 2.6 ± 0.4 |
|  |  |  | 24 h | 35.8 ± 2.3 | 31.7 ± 1.5 | 0.8 ± 0.4 | 0.6 ± 0.2 |
| E4 | Females | Young | Naïve | 18.1 ± 3.6 | See Fig. 5 | 1.0 ± 0.3 | 0.5 ± 0.2 |
|  |  |  | 1.5 h | 28.6 ± 1.6 | 17.3 ± 2.0 | 7.0 ± 0.5 | 3.8 ± 0.5 |
|  |  |  | 24 h | 30.6 ± 1.0 | 12.3 ± 0.9 | 3.7 ± 0.9 | 1.7 ± 0.3 |
|  |  | Middle-Aged | Naïve | 30.0 ± 3.6 | See Fig. 5 | 0.1 ± 0.2 | 0.2 ± 0.1 |
|  |  |  | 1.5 h | 27.1 ± 1.7 | 22.4 ± 1.5 | 1.0 ± 0.2 | 1.1 ± 0.4 |
|  |  |  | 24 h | 29.4 ± 1.8 | 26.2 ± 1.7 | 0.0 | 0.3 ± 0.2 |
|  | Males | Young | Naïve | 33.6 ± 2.8 | See Fig. 5 | 2.4 ± 0.7 | 0.9 ± 0.3 |
|  |  |  | 1.5 h | 21.8 ± 2.6 | 15.2 ± 1.7 | 2.8 ± 0.9 | 1.6 ± 0.5 |
|  |  |  | 24 h | 23.6 ± 4.7 | 13.8 ± 4.7 | 2.8 ± 1.5 | 1.7 ± 1.0 |
|  |  | Middle-Aged | Naïve | 28.4 ± 2.7 | See Fig. 5 | 0.5 ± 0.3 | 0.3 ± 0.2 |
|  |  |  | 1.5 h | 30.6 ± 2.8 | 23.1 ± 2.4 | 2.6 ± 0.6 | 1.7 ± 0.4 |
|  |  |  | 24 h | 29.6 ± 1.5 | 23.4 ± 2.1 | 0.3 ± 0.2 | 0.5 ± 0.1 |

^1^ All data are measured in counts and presented as averages ± SEM.

**Supplemental Figure 1**. The number of cFos+ cells in the CA3. **A)** cFos in the CA1 of young females. There was an effect of time (*p* < 0.0001, *F*(2,21) = 15.11). E3 euthanized at 1.5 h had more cFos than naïve E3 mice (*p* = 0.032) and E4 at 1.5 h had more cFos than naïve (*p* = 0.002) and 24 h (*p* = 0.004). **B)** cFos in the CA1 of young males. There was an effect of time (*p* = 0.0402). Post hoc testing did not reveal any significant differences in individual genotypes. **C)** cFos in the CA1 of middle-aged females. There were no effects. **D)** cFos in the CA1 of middle-aged males. There was an effect of time (*p* < 0.0001) and genotype (*p* < 0.0001). E2 mice euthanized at 1.5 h had more cFos than naïve (*p* = 0.0008) and 24 h (*p* < 0.0001); E3 mice euthanized at 1.5 h also had more cFos than naïve (*p* = 0.008) and 24 h (*p* = 0.009). E) Representative images of the CA3. White arrows indicate cFos+ cells, yellow arrows indicate 53BP1+ cells, the red arrow indicates a co-localized signal. **p* < 0.05, ***p* < 0.01, ****p* < 0.001, ^*p* < 0.0001.
